# Registry-based estimation of cardiac event–free survival in congenital heart disease complicated by pulmonary hypertension: A nationwide registry study from Japan

**DOI:** 10.1016/j.ijcchd.2026.100679

**Published:** 2026-04-19

**Authors:** Taku Ishii, Tatsuhiko Anzai, Keiko Uchida, Susumu Hosokawa, Naofumi F. Sumitomo, Hidekazu Ishida, Keiichi Hirono, Jun Muneuchi, Ayako Chida-Nagai, Ryo Inuzuka, Hirofumi Sawada, Sayo Suzuki, Jun Maeda, Hisaaki Aoki, Lisheng Lin, Takashi Murakami, Yusuke Nakano, Tatsuya Onishi, Takuya Wakamiya, Kei Inai, Shinichi Takatsuki, Atsushi Yao, Shigetoyo Kogaki, Hiroyuki Fukushima, Yuichi Tamura, Kunihiko Takahashi, Hiroyuki Yamagishi, Shozaburo Doi

**Affiliations:** a Department of Pediatrics, Institute of Science Tokyo, 1-5-45 Yushima, Bunkyo-ku, Tokyo, 113-8519, Japan; b Department of Biostatistics, M&D Data Science Center, Institute of Science Tokyo, 1-5-45 Yushima, Bunkyo-ku, Tokyo, 113-8519, Japan; c Department of Physiology, Tokyo Medical University, 6-7-1 Nishishinjuku, Shinjuku-ku, Tokyo, 160-0023, Japan; d Department of Pediatrics, Japanese Red Cross Musashino Hospital, 1-26-1 Kyonan-cho, Musashino, Tokyo, 180-0023, Japan; e Department of Pediatrics, Keio University, 35 Shinanomachi, Shinjuku-ku, Tokyo, 160-8582, Japan; f Department of Pediatrics, The University of Osaka Graduate School of Medicine, 2-2 Yamadaoka, Suita, Osaka, 565-0871, Japan; g Department of Pediatrics, Toyama University Hospital, 2630 Sugitani, Toyama, 930-0194, Japan; h Department of Pediatrics, Japan Community Health Care Organization Kyushu Hospital, 1-8-1 Kishinoura, Yahatanishi-ku, Kitakyushu, Fukuoka, 806-8501, Japan; i Department of Pediatrics, Hokkaido University, Kita 14-jo Nishi 5-chome, Kita-ku, Sapporo, Hokkaido, 060-8648, Japan; j Department of Pediatrics, The University of Tokyo, 7-3-1 Hongo, Bunkyo-ku, Tokyo, 113-8655, Japan; k Department of Pediatrics, Mie University, 2-174 Edobashi, Tsu, Mie, 514-8507, Japan; l Department of Cardiology, Fukuoka Children's Hospital, 5-1-1 Kashii-teruha, Higashi-ku, Fukuoka, 813-0017, Japan; m Department of Cardiology, Tokyo Metropolitan Children's Medical Center, 2-8-29 Musashidai, Fuchu, Tokyo, 183-8561, Japan; n Department of Cardiology, Osaka Women's and Children's Hospital, 840 Murodo-cho, Izumi, Osaka, 594-1101, Japan; o Department of Pediatric Cardiology, Ibaraki Children's Hospital, 3-3-1 Futabadai, Mito, Ibaraki, 311-4145, Japan; p Department of Child Health, Institute of Medicine, University of Tsukuba, 2-1-1 Amakubo, Tsukuba, Ibaraki, 305-8576, Japan; q Department of Pediatrics, Yokohama City University, 3-9 Fukuura, Kanazawa-ku, Yokohama, Kanagawa, 236-0004, Japan; r Department of Pediatric Cardiology, National Hospital Organization Shikoku Medical Center for Children and Adults, 2-1-1 Senyu-cho, Zentsuji, Kagawa, 765-8507, Japan; s Department of Cardiology, Kanagawa Children's Medical Center, 2-138-4 Mutsukawa, Minami-ku, Yokohama, Kanagawa, 232-8555, Japan; t Department of Pediatric Cardiology and Adult Congenital Cardiology, Tokyo Women's Medical University, 8-1 Kawada-cho, Shinjuku-ku, Tokyo, 162-8666, Japan; u Department of Pediatrics, Toho University Omori Medical Center, 6-11-1 Omorinishi, Ota-ku, Tokyo, 143-8541, Japan; v Division for Health Service Promotion, The University of Tokyo, 7-3-1 Hongo, Bunkyo-ku, Tokyo, 113-8655, Japan; w Department of Pediatrics and Neonatology, Osaka General Medical Center, 3-1-56 Bandai-higashi, Sumiyoshi-ku, Osaka, 558-8558, Japan; x Tokyo Dental College Ichikawa General Hospital, 5-11-13 Sugano, Ichikawa, Chiba, 272-0824, Japan; y Department of Cardiology and Medical Education, International University of Health and Welfare, 1-4-3 Mita, Minato-ku, Tokyo, 108-8329, Japan; z Tokyo Metropolitan Children's Medical Center, 2-8-29 Musashidai, Fuchu, Tokyo, 183-8561, Japan

**Keywords:** Congenital heart disease, Pulmonary hypertension, Nationwide registry study, Cardiac event-free survival, Prognostic model, Risk prediction

## Abstract

**Background:**

Pulmonary hypertension (PH) is a major determinant of outcomes in congenital heart disease (CHD), yet tools for individualized prognostic estimation are limited. This study aimed to develop an exploratory, clinically oriented prediction model for estimating cardiac event–free survival in patients with CHD-PH.

**Methods:**

Data from the nationwide Japanese Association of CHD-PH Registry were analyzed in a retrospective cohort derived from a prospectively maintained registry. Cardiac event–free survival was evaluated using Cox proportional hazards models incorporating prespecified, routinely available clinical and hemodynamic variables. Cardiac events were defined as death, transplantation, clinical worsening requiring treatment escalation, or PH-related hospitalization.

**Results:**

A total of 224 patients were included, and 23 experienced 29 cardiac events during a median follow-up of 1.5 years. These included 7 cardiovascular deaths, 1 lung transplantation, and 1 atrial septostomy, 13 cases of clinical worsening. The cardiac event–free survival rates were 94.0% and 86.2% at 1 and 2 years, respectively. In multivariable analysis, trisomy 21 (hazard ratio [HR] 3.63), elevated pulmonary vascular resistance index (PVRI; HR 13.2), and elevated central venous pressure (CVP; HR 3.27) were associated with worse outcomes. These associations were consistent in sensitivity and subgroup analyses. Model-based estimates demonstrated risk gradients across clinically relevant profiles and were incorporated into a prototype prediction tool.

**Conclusions:**

An exploratory pragmatic prediction model for cardiac event–free survival in CHD-PH was developed using nationwide registry data. These findings support the feasibility of a registry-based prognostic framework; however, further validation is required before clinical application.

## Highlights

• Nationwide registry-based analysis provides insights into short-term outcomes in CHD-PH.
• Cardiac event–free survival reached 94.0% at 1 year and 86.2% at 2 years.
• Trisomy 21, elevated PVRI, and elevated CVP were associated with cardiac events.
• Model-based estimates illustrate risk gradients across clinical profiles.
• Prototype framework demonstrates feasibility of registry-based prognostic estimation.

## Graphical abstract

**Figure:**
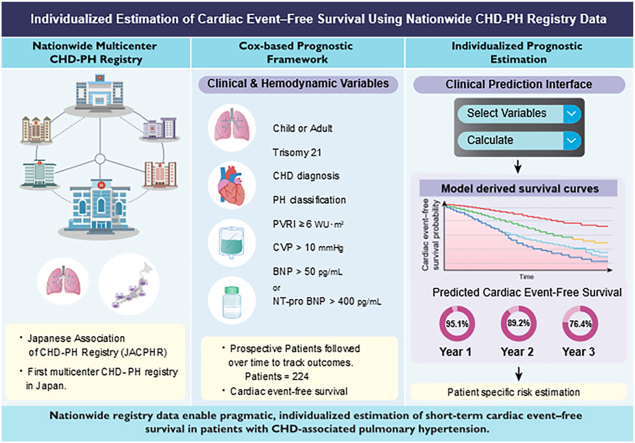

## 1 Introduction

Congenital heart disease (CHD) is among the most common congenital anomalies, affecting approximately 1% of live births [1,2]. Advances in pediatric cardiology and cardiovascular surgery have markedly improved survival outcomes among patients with CHD [3,4], and an increasing number of such patients reach adulthood in Japan [5]. Consequently, the long-term management of CHD has become an increasingly important clinical and societal concern. Pulmonary hypertension (PH) in patients with CHD is a major determinant of long-term outcomes [6,7].

In recent years, therapeutic options for PH have expanded [[8], [9], [10]], which have improved care increased complexity to the management of CHD complicated by PH (CHD-PH). Although several Western registries have examined prognostic factors in pediatric and adult populations with PH [[11], [12], [13]], these studies included few CHD-PH cases, and no multicenter prognostic data specific to CHD-PH in Japan are available. Furthermore, although current PH guidelines provide structured risk-stratification frameworks [14], these classifications were developed more broadly for pulmonary arterial hypertension (PAH) and do not fully address the unique anatomy, physiology, and clinical trajectories of CHD-PH.

Registry-based prognostic research is essential; however, the composition of registry cohorts, treatment strategies, and clinical practices inevitably evolve over time, potentially altering the relative importance of prognostic factors. Although newly accumulated information from expanding registries should be rapidly incorporated into risk stratification, existing risk models [15] are inherently static and cannot easily integrate new knowledge. Moreover, most registry-derived risk models estimate mortality risk using a cumulative or additive framework, in which predefined risk factors are comprehensively assessed. However, in heterogeneous disease entities such as CHD-PH, more individualized risk assessment may be required, accounting for the relative contribution of each factor and the potential impact of coexisting risk features on prognosis.

Against this background, this study aimed to develop an exploratory, registry-based approach for generating prognostic estimates and to present a prototype framework for individualized prediction in patients with CHD-PH. Using data from a nationwide multicenter registry in Japan, this study sought to construct a flexible model that can accommodate evolving real-world data and illustrate potential approach to assessment in daily practice.

## 2 Methods

### 2.1 Study design and registry

This study was a retrospective cohort analysis derived from a prospectively maintained nationwide multicenter registry, the Japanese Association of CHD-PH Registry (JACPHR), involving 49 institutions providing care for patients with CHD across Japan. The registry was initiated in August 2021 and was designed to collect detailed clinical, hemodynamic, and outcome data on patients with CHD-PH. The study was conducted in accordance with the principles of the Declaration of Helsinki. The protocol was approved by the Medical Research Ethics Committee of the Institute of Science Tokyo (approval number M2017-325), which served as the coordinating center of the registry. In addition, all participating institutions obtained approval from their respective institutional review boards. Written informed consent was obtained from all patients or their legal guardians prior to enrollment. The study was registered with the UMIN Clinical Trials Registry (UMIN-CTR; UMIN000032366).

### 2.2 Registry eligibility criteria

The main inclusion criteria for the JACPHR registry include the presence of CHD, irrespective of the anatomical complexity or repair status, complicated by PH. PH is defined as either a mean pulmonary arterial pressure (mPAP) > 20 mmHg or a pulmonary vascular resistance index (PVRI) ≥ 3 Wood units·m^2^ (WU·m^2^). Patients with segmental PH and those with Fontan circulation were eligible under predefined conditions. Specifically, PH had to be documented at least 6 months before or after any surgical or catheter-based intervention that could affect pulmonary hemodynamics. Detailed eligibility criteria were described previously [16] and are summarized in the Supplementary Method 1.

### 2.3 Study population for the present analysis

**Fig. 1:**
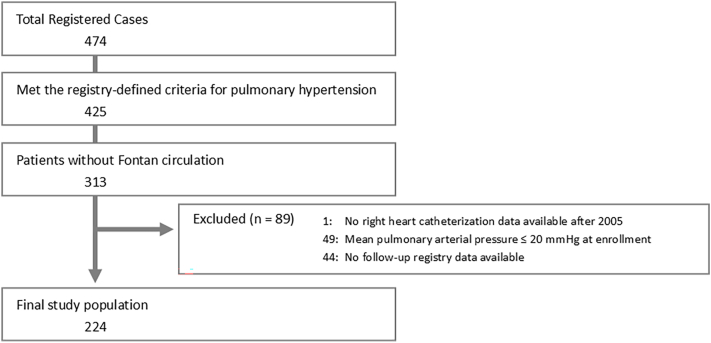
Patient selection.

For the present analysis, patients registered between August 2021 and March 2025 who fulfilled the JACPHR eligibility criteria were screened. Patients with Fontan circulation were excluded. Although the JACPHR registry includes patients with a previous history of PH who later exhibited clinical improvement, this study was restricted to patients who met all of the following additional criteria: availability of right-heart catheterization data obtained in 2005 or later, mPAP >20 mmHg at the most recent catheterization performed before or during registry enrollment, and at least one prospective follow-up registration after enrollment. After applying these criteria, 224 patients were included in the final study cohort (Fig. 1).

### 2.4 Outcome definition and follow-up

The primary outcome was cardiac event–free survival. Overall survival was analyzed as secondary outcome. Cardiac event-free survival was defined as the time from registry enrollment to the first occurrence of any PH-related cardiac event. Cardiac events included cardiovascular death (defined as PH-related death or death due to right heart failure), lung or heart–lung transplantation, atrial septostomy or surgical creation of an interatrial communication, clinical worsening of PH symptoms (defined as worsening of the World Health Organization functional class and/or a clinical condition requiring escalation of PH-specific therapy), and hospitalization because of PH. Non-cardiovascular deaths were excluded from the composite outcome to maintain etiologic consistency. Follow-up data were recorded at the time of any cardiac event and, in the absence of events, were updated at least annually. Event-free patients were censored at the last available follow-up. In the analysis of cardiac event–free survival, deaths not classified as cardiovascular death were treated as censoring events at the time of death. Outcomes were assessed prospectively through scheduled registry updates.

### 2.5 Candidate predictors and variable definitions

In this study, a multivariable prognostic analysis was performed using data from a prospectively maintained registry, and the results formed the basis for the development of a prediction model for cardiac event–free survival in patients with CHD-PH. Candidate predictors were selected a priori based on clinical relevance, pathophysiological plausibility, and routine availability in daily practice [14,17]. Baseline variables included the age category (0–17 vs. ≥18 years), trisomy 21 status, and clinical PH classification [18,19].

At enrollment, PH was classified according to the classification proposed at the 2018 World Symposium on PH [20] (Supplementary Method 3). For prognostic modeling performed here, PH categories were further consolidated into four clinically meaningful groups. Patients with CHD-associated PAH (group 1) were subdivided into those “with shunt” (Eisenmenger syndrome, left-to-right shunt lesions, or coincidental PH) and those “without shunt” (repaired CHD). PH due to left-heart disease (group 2), including conditions such as pulmonary vein stenosis following repair of total anomalous pulmonary venous return or left-sided atrioventricular valve dysfunction, was analyzed as a separate category, given its distinct pathophysiology and management strategies [16,21]. All remaining etiologies, including lung disease–associated PH, segmental PH, and other rare forms, were grouped as “other.”

Additional predictors included PVRI as an indicator of pulmonary vascular disease severity, central venous pressure (CVP) as a surrogate of right ventricular function, and B-type natriuretic peptide (BNP) as a general marker of heart failure severity; these parameters are routinely assessed and associated with PH outcomes. Cutoff values were predefined based on clinical relevance. A high PVRI was defined using a threshold commonly applied in pediatric CHD-associated PAH when considering operability [18], set at PVRI ≥6 WU·m^2^. A high BNP level was defined as > 50 pg/mL, in accordance with contemporary PH guidelines [14]. For patients with unavailable BNP data, an N-terminal BNP (NT-proBNP) level >400 pg/mL was used as a surrogate threshold. A high CVP is generally considered elevated when it exceeds the normal range in pediatric and young adult populations, using a cutoff of >10 mmHg [22,23]. To address the potential limitations of dichotomization, restricted cubic spline analyses were performed for PVRI and CVP. When the data-driven inflection points differed from the prespecified clinical thresholds, supplementary analyses using alternative cutoffs were conducted.

### 2.6 Statistical analysis and prognostic modeling

Cox proportional hazards models were used to evaluate associations between candidate predictors and cardiac event–free survival. Analyses were performed using complete-case data without imputation. Model assumptions and fit were assessed using standard diagnostic methods. Given the limited number of events, internal validation was performed using bootstrapping resampling, and uniform shrinkage was applied to potential overfitting. Prespecified subgroup analyses were conducted according to age category, CHD subtype, and a sensitivity cohort restricted to patients who underwent right heart catheterization within 1 year prior to registry enrollment. Based on the fitted Cox model, predicted cardiac event-free survival at 1, 2, and 3 years was estimated for predefined clinical profiles, and a prototype clinical prediction tool was constructed. Detailed procedures are provided in the Supplementary Method 4.

All statistical analyses were performed using Stata software (StataCorp, College Station, TX, USA) [24]. Predicted survival estimates were generated using standard post-estimation procedures for Cox models, implemented using the survci command. A two-sided p value < 0.05 was considered statistically significant.

## 3 Results

### 3.1 Patient characteristics (Table 1)

A total of 224 patients with CHD-PH were included in the final analysis (Fig. 1). Their baseline characteristics are summarized in Table 1. The cohort did not differ by sex and were relatively young (median age, 11.7 years). Approximately half of the patients had a systemic underlying disease, with trisomy 21 being the most common (25%). In the clinical PH classification, postoperative PH was most common, whereas post-tricuspid shunt lesions were the most prevalent in major CHD diagnoses. Upon registration, approximately 70% of the patients were receiving PH-targeted therapy, and nearly half of the entire cohort received combination therapy.

**Table 1:** Baseline characteristics of the patients.

**Total cases**		224	
**Sex, N (%)**			
Male		104	(46.4)
Female		120	(53.6)
**Age at registration, years, median (IQR)**		11.7	(6.0–21.2)
0–17 years, N (%)		157	(70.1)
≥18 years, N (%)		67	(29.9)
**Follow-up duration from enrollment, years, median (IQR)**		1.5	(1.1–2.3)
**Comorbidities, N (%)**			
Underlying systemic disease		108	(48.2)
Trisomy 21		56	(25.0)
22q11.2 deletion syndrome		21	(9.4)
Left atrial isomerism		6	(2.7)
Other		25	(11.2)
Airway complications		36	(16.1)
**Major cardiac diagnosis, N (%)**			
Pre-tricuspid shunt		53	(23.7)
Post-tricuspid shunt		79	(35.3)
Complex or other CHD		92	(41.1)
**PH classification (WSPH 2018), N (%)**		**N**	**(%)**
Group 1	Eisenmenger syndrome	15	(6.7)
	Large left-to-right shunt	11	(4.9)
	Coincidental PH	27	(12.1)
	Postoperative PH	95	(42.4)
Group 2	Left-heart disease	23	(10.3)
Group 5	Segmental PH	29	(12.9)
Others		24	(10.7)
**Medications for PH at registration, N (%)**			
No medication		73	(32.6)
Monotherapy		47	(21.0)
Combination therapy		104	(46.4)
**Right heart catheterization (RHC) findings**			
Time from RHC to registration, months, median (IQR) (N = 224)		16.2	(3.6–42.6)
Mean PAP (mmHg) (N = 224)		28	(24–40)
PVRI (WU·m^2^) (N = 206)		4.7	(3.5–8.9)
CVP (mmHg) (N = 218)		7	(5–9)
**Laboratory findings**			
Time from laboratory test to registration, months, median (IQR) (N = 212)		1.4	(0.0–6.7)
BNP, pg/mL, median (IQR) (N = 175)		29	(14–64)
NT-proBNP, pg/mL, median (IQR) (N = 48)		180	(72–403)
Total bilirubin, mg/dL, median (IQR) (N = 206)		0.6	(0.4–0.8)
Creatinine, mg/dL, median (IQR) (N = 212)		0.43	(0.31–0.62)

BNP, B-type natriuretic peptide; CHD, congenital heart disease; NT-proBNP, N-terminal pro-B-type natriuretic peptide; PAP, pulmonary artery pressure; PH, pulmonary hypertension; PVRI, pulmonary vascular resistance index; RHC, right heart catheterization; WSPH 2018, World Symposium on Pulmonary Hypertension (2018); WU, Wood units.

### 3.2 Survival outcomes and prognostic analysis

**Fig. 2:**
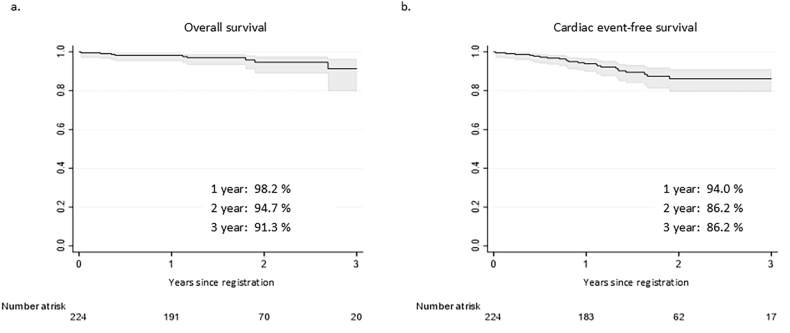
**Overall survival and cardiac event–free survival in patients with CHD-PH** Kaplan–Meier curves for overall survival (a) and cardiac event–free survival (b) in patients with CHD-PH, with one-, two-, and three-year estimates and numbers at risk; shaded areas denote 95% confidence intervals. CHD, congenital heart disease; CI, confidence interval; PH, pulmonary hypertension.

During a median follow-up period of 1.5 (interquartile range, 1.1–2.3) years, 9 deaths were observed. In addition, a total of 29 cardiac events occurred in 23 patients. Among these events, there were 7 cardiovascular deaths (4 PH-related and 3 due to right heart failure), 1 lung transplantation, 1 atrial septostomy, 13 cases of clinical worsening of PH, and 7 hospitalizations due to PH. Survival outcomes derived from the registry are shown in Fig. 2. The 1-, 2-, and 3-year overall survival rates were 98.2%, 94.7%, and 91.3%, respectively (Fig. 2a). The corresponding 1-, 2-, and 3-year cardiac event–free survival rates were 94.0%, 86.2%, and 86.2% (Fig. 2b). The distribution of patients with cardiac events according to PH classification and underlying cardiac diagnosis is presented in Supplementary Table 1.

A Cox proportional hazards analysis was conducted to evaluate the prognostic performance of models incorporating clinically relevant variables selected a priori, including age category, trisomy 21 status, PH classification, PVRI, CVP, BNP, and PH-targeted pharmacological therapy (Table 2). The analysis was based on complete-case data, including 190 patients and 21 cardiac events. Two models were examined: Model 1 included six prespecified covariates, and Model 2 additionally incorporated PH-targeted pharmacological therapy. In Model 1, elevated PVRI (hazard ratio [HR] 13.2, 95% CI 3.50–49.4), elevated CVP (HR 3.27, 95% CI 1.14–9.39), and trisomy 21 (HR 3.63, 95% CI 1.24–10.6) were significantly associated with worse cardiac event–free survival. Similar results were observed in Model 2. Internal validation using bootstrapping indicated modest optimism in model performance. Accordingly, uniform shrinkage was applied, resulting in attenuation of regression coefficients while preserving the overall pattern of associations. The overall model fit was as follows: Model 1, LR χ^2^(8) = 31.48 (p = 0.0001); Model 2, LR χ^2^(10) = 32.9 (p = 0.0003).

**Table 2:** Multivariable Cox Proportional Hazards analysis of cardiac event–free survival in patients with CHD-PH.

	**Model 1**		**Model 2**	
	**HR (95% CI)**	**Shrinkage-adjusted HR (95% CI)**	**HR (95% CI)**	**Shrinkage-adjusted HR (95% CI)**
**Age at registration**				
≥18 years	0.85 (0.30–2.44)	0.89 (0.41–1.94)	0.75 (0.26–2.21)	0.82 (0.39–1.74)
**Comorbidities**				
Trisomy 21	**3.63 (1.24**–**10.6)**	**2.61 (1.18**–**5.84)**	**3.61 (1.23**–**10.64)**	**2.44 (1.15**–**5.19)**
**PH classification**				
With LR shunt	Ref	Ref	Ref	Ref
Without LR shunt	2.01 (0.71–5.69)	1.69 (0.78–3.63)	2.01 (0.71–5.74)	1.63 (0.79–3.38)
Left-heart disease	1.89 (0.21–16.7)	1.61 (0.32–8.18)	2.05 (0.23–18.17)	1.65 (0.36–7.53)
Other	1.43 (0.26–7.95)	1.31 (0.36–4.70)	1.22 (0.21–6.97)	1.15 (0.34–3.87)
**PVRI at registration**				
PVRI >6 WU·m^2^	**13.2 (3.50**–**49.4)**	**6.83 (2.55**–**18.26)**	**11.61 (3.03**–**44.53)**	**5.51 (2.16**–**14.05)**
**CVP at registration**				
CVP >10 mmHg	**3.27 (1.14**–**9.39)**	**2.43 (1.10**–**5.32)**	**3.43 (1.21**–**9.67)**	**2.36 (1.14**–**4.85)**
**BNP at registration**^a^				
BNP >50 pg/mL	1.44 (0.56–3.69)	1.31 (0.65–2.66)	1.55 (0.58–4.15)	1.36 (0.69–2.69)
**Medication**				
**No medication**			Ref	Ref
**Monotherapy**			1.17 (0.25–5.50)	1.12 (0.38–3.28)
**Combination therapy**			1.89 (0.59–6.09)	1.56 (0.69–3.52)

Results of the Cox proportional hazards analysis of cardiac event–free survival in 190 patients with CHD-PH included in the complete-case analysis. Model 1 included six covariates (8 degrees of freedom; LR χ^2^(8) = 31.48, p = 0.0001), and Model 2 included seven covariates, additionally incorporating pulmonary hypertension–targeted pharmacological therapy as a covariate (10 degrees of freedom; LR χ^2^(10) = 32.9, p = 0.0003). Degrees of freedom reflect dummy variables for categorical covariates. A total of 21 cardiac events occurred during 307 patient-years of observation. Both original hazard ratios and shrinkage-adjusted hazard ratios are presented. Shrinkage factors were 0.746 for Model 1 and 0.696 for Model 2.

BNP, B-type natriuretic peptide; CHD, congenital heart disease; CI, confidence interval; CVP, central venous pressure; HR, hazard ratio; NT-proBNP, N-terminal pro-B-type natriuretic peptide; PH, pulmonary hypertension; PVRI, pulmonary vascular resistance index; WU, Wood units.

Bold values indicate p < 0.05.

a BNP at registration: NT-proBNP values were used when BNP was not available (cutoff >400 pg/mL).

Additional analyses using restricted cubic splines demonstrated a nonlinear association between both PVRI and CVP and the risk of cardiac events (Supplementary Fig. 1). For CVP, the risk appeared to increase around 10 mmHg, supporting the prespecified cutoff; therefore, no additional cutoff validation was performed. In contrast, PVRI showed a steep increase in hazard around 5 WU·m^2^, and a sensitivity analysis using an alternative cutoff of PVRI ≥5 WU·m^2^ was conducted. The direction and magnitude of associations were largely consistent with the primary analysis in both Model 1 and Model 2 (Supplementary Table 2). Exploratory subgroup analyses were performed stratified by age category (children and adolescents vs adults), CHD subtype (pre-tricuspid vs post-tricuspid shunt), a subgroup restricted to patients with CHD-PH without residual shunt, representing a relatively homogeneous population, and a cohort restricted to patients who underwent right heart catheterization within 1 year prior to enrollment (Supplementary Table 3). Due to the limited number of events, models were restricted to three covariates (trisomy 21, PVRI, and CVP). In adult patients, no events occurred among those with trisomy 21, precluding estimation of the hazard ratio. Although statistical significance varied across subgroups, likely due to limited sample size, the direction of associations was generally consistent, with elevated hazard ratios observed for all three variables. These analyses were exploratory and not powered for definitive inference.

### 3.3 Development of a cardiac event prediction model

**Fig. 3:**
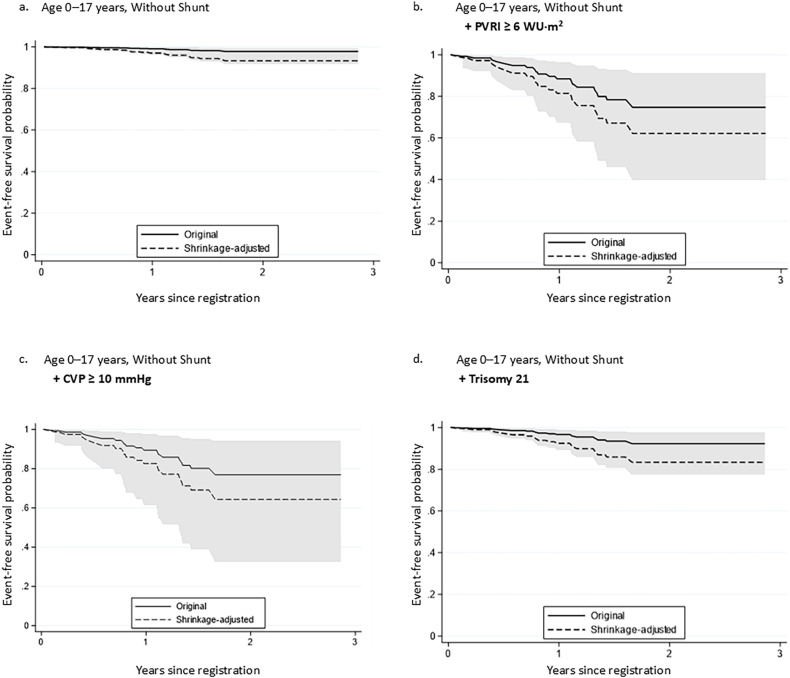
**Representative adjusted cardiac event–free survival curves derived from the Cox proportional hazards model** Predicted cardiac event–free survival curves were generated from the Cox proportional hazards model for four representative clinical profiles. All panels represent pediatric patients (age 0–17 years) without residual shunt. Solid lines indicate the original model–based predicted survival curves, shaded areas represent 95% confidence intervals (CIs), and dashed lines indicate shrinkage-adjusted predicted survival curves. The four panels show the following profiles: (a) baseline profile (without shunt, no additional risk factors), (b) elevated pulmonary vascular resistance index (PVRI ≥6 WU·m^2^), (c) elevated central venous pressure (CVP ≥10 mmHg), and (d) trisomy 21. CI, confidence interval; CVP, central venous pressure); PVRI, pulmonary vascular resistance index.

Using the previously described Cox proportional hazards model, adjusted survival probabilities and corresponding 95% CIs at 1 and 2 years were estimated for predefined clinical profiles to illustrate how the model differentiates risk across clinically relevant scenarios. Table 3 presents representative predictions for selected clinical profiles that were relatively common within the study cohort. Differences in predicted survival were observed across these profiles, with consistent influences of elevated PVRI, elevated CVP, and trisomy 21 compared with profiles without these risk factors. Both original and shrinkage-adjusted cardiac event–free survival estimates are presented in Table 3. To illustrate how the model differentiates risk across clinically relevant scenarios, Fig. 3 presents adjusted survival curves for representative profiles in pediatric patients without residual shunt, demonstrating the impact of individual risk factors, including elevated PVRI, elevated CVP, and trisomy 21. The distribution of patients across observed predictor profiles is summarized in Supplementary Table 4. A total of 54 distinct profiles were identified among the study cohort, and many profiles included only a small number of patients. These findings indicate that estimates for sparsely represented profiles should be interpreted with caution.

**Table 3:** Predicted 1- and 2-year cardiac event-free survival across representative clinical profiles based on the Cox Proportional Hazards model.

PH classification	Background	Clinical data	Estimated 1-year CEFS (%, [95% CI])	Shrinkage-adjusted 1-year CEFS (%)	Estimated 2-year CEFS (%, [95% CI])	Shrinkage-adjusted 2-year CEFS (%)	N
Without shunt	0–17 years	–	99.1 (96.2–99.8)	97.0	97.8 (91.7–99.4)	93.3	13
Without shunt	0–17 years	High PVRI	88.4 (67.2–96.3)	81.4	74.7 (39.7–91.2)	62.2	6
Without shunt	0–17 years	High CVP	97.0 (87.7–99.3)	90.9	93.0 (74.3–98.3)	80.2	7
Without shunt	≥18 years	–	99.2 (96.0–99.8)	97.4	98.1 (91.4–99.6)	94.0	7
Without shunt	0–17 years Trisomy 21	–	96.7 (89.3–99.0)	92.4	92.3 (77.4–97.5)	83.4	19
With shunt	0–17 years	–	99.5 (98.0–99.9)	98.2	98.9 (95.2–99.8)	96.0	7
Left-heart	0–17 years	–	99.1 (92.8–99.9)	95.0	98.0 (84.4–99.7)	88.9	8

Predicted cardiac event-free survival (CEFS), as defined in the main text, at 1 and 2 years was estimated from the Cox proportional hazards model for representative clinical profiles. Shrinkage-adjusted CEFS estimates were obtained by applying a uniform shrinkage factor to the regression coefficients. Values are presented as percentages with 95% confidence intervals for the original model. “Clinical data” indicates the presence of clinically relevant risk factors, including elevated pulmonary vascular resistance index (PVRI >6 WU·m^2^) and elevated central venous pressure (CVP >10 mmHg). No profiles included elevated BNP. Age groups were defined as 0–17 years and ≥18 years. N indicates the number of patients in the original dataset corresponding to each profile.

CEFS, cardiac event-free survival; CI, confidence interval; CVP, central venous pressure; PH, pulmonary hypertension; PVRI, pulmonary vascular resistance index; WU, Wood units.

### 3.4 Illustrative framework for profile-based risk estimation

Finally, based on the model-derived estimates, we developed an illustrative framework to show how profile-based estimates of cardiac event–free survival can be derived from the model. Adjusted survival probabilities at 1, 2, and 3 years, along with corresponding 95% CIs were pre-computed for all possible combinations of predictor variables and organized into a structured reference dataset. This framework organizes pre-computed estimates for combinations of clinical variables and demonstrates how corresponding predicted cardiac event–free survival values and adjusted survival curves can be displayed in both numerical and graphical formats. This framework enables intuitive visualization of the impact of different risk factor combinations on expected outcomes. Supplementary Fig. 2 illustrates the structure of this prototype tool and the reference sheets used to provide patient-specific prognostic predictions for individuals with CHD-PH. However, as demonstrated in Supplementary Table 4, the number of patients contributing to each profile varied substantially, and some profiles were sparsely represented or not observed in the study cohort. Therefore, predictions for such profiles should be interpreted with caution, as they may rely heavily on model extrapolation.

## 4 Discussion

### 4.1 Principal findings

This nationwide registry-based study evaluated outcomes from the time of enrollment and introduced a clinically oriented prognostic framework for patients with CHD-PH using JACPHR data. Given the low death rate during the relatively short follow-up period, mortality alone was not suitable as the primary endpoint; therefore, cardiac event–free survival was adopted as the outcome measure. Clinically, this approach is appropriate as therapeutic decisions in CHD-PH are typically made well before death, and estimation of near-term freedom from clinically meaningful cardiac events may be more relevant to routine clinical management compared to mortality alone. Within this exploratory framework, elevated PVRI, and elevated CVP, and trisomy 21 were associated with differences in cardiac event–free survival. These findings suggest that hemodynamic burden and patient-related factors may contribute to short-term risk stratification in this heterogeneous population. Importantly, this study should be considered as a proof-of-concept demonstrating the feasibility of constructing a registry-based prognostic model and a corresponding prediction tool using routinely available clinical variables. Given the limited sample size, heterogeneity of the study population, and lack of external validation, the proposed framework and tool are exploratory in nature and require further validation before clinical application.

### 4.2 Comparison with previous studies

Although direct comparisons across studies are restricted by differences in patient populations and follow-up duration, the short-term outcomes in this cohort appear favorable compared with previously reported CHD-PH reports [12,13,25,26]. Survival was comparable to that in contemporary Japanese adult PAH cohorts [27], and aligns with prior registry data suggesting similar or better outcomes in CHD-associated PAH [6,12,28]. The relatively favorable prognosis of PAH in Japan has been attributed, at least in part, to early diagnosis and widespread access to PH-targeted therapies under the universal health insurance system [29,30].

PVRI showed a strong association with differences in cardiac event–free survival. Assessing CHD-PH severity by mPAP is challenging due to the influence of shunt physiology and left-sided filling pressures. PVRI provides a more direct measure of pulmonary vascular disease across phenotypes and correlates with long-term prognosis [13,26]. Consequently, despite being consensus-based, the established 6 WU·m^2^ threshold [18] was utilized in this study for pragmatic reasons. Spline analyses suggested a nonlinear increase in risk around 5 WU·m^2^; however, findings were exploratory, and sensitivity analyses using a ≥5 WU·m^2^ cutoff yielded consistent results. Similarly, CVP showed increased risk around 10 mmHg, supporting the clinical relevance of this threshold [14].

Trisomy 21 was identified as a factor associated with cardiac event–free survival. This subgroup was predominantly pediatric (53/56 patients), which likely explains the absence of events among the few adult cases. While the 35.7% prevalence in our pediatric cohort is consistent with the known association between trisomy 21 and CHD-PH [31,32], its lower representation in adult registries (approximately 13%) [25] may reflect survival bias or limited data. These findings suggest that trisomy 21 may be associated with increased cardiac event risk in pediatric CHD-PH [32,33], whereas its prognostic significance in adults requires further study. This high prevalence should be considered when interpreting the overall results.

Age was not associated with short-term outcomes after adjustment. While pediatric PAH patients often present with more severe disease at diagnosis, their post-treatment prognosis is comparable to that of adults [34,35]. Our findings are consistent with this observation in CHD-PH, suggesting that short-term prognosis may be driven primarily by pathophysiology and hemodynamic burden rather than age. Although BNP showed a relatively high HR, it did not reach statistical significance, likely due to limited statistical power from the modest number of patients and events. Similarly, PH-targeted therapy was not associated with improved cardiac event-free survival. The observed trend toward higher HRs with treatment escalation likely reflects confounding by indication, whereby therapy is preferentially administered to patients with more severe disease [16]. Such inherent challenges in registry-based comparisons, together with the predominance of patients with repaired CHD rather than Eisenmenger syndrome, may explain the absence of an apparent treatment effect. While the benefits of PH-targeted therapy are established in Eisenmenger syndrome [26,36], evidence in repaired CHD remains limited [6]. In addition, the relatively small sample size and short follow-up may have reduced the ability to detect treatment effects, warranting further investigation. Moreover, time-varying effects of treatment were not assessed, which may have further influenced these findings.

### 4.3 Clinical implications

The primary objective of this study was not to establish a definitive or fully optimized prediction model but to explore a practical, hypothesis-generating approach for estimating near-term cardiac event risk using information routinely available in clinical practice. In real-world care, catheterization data are often not contemporaneous, and advanced imaging may not be available for guideline-based risk stratification. JACPHR registry data reflect the information that clinicians typically have upon assessment; thus, the resulting prognostic estimates are intended to be aligned with everyday clinical decision-making. The framework shown in Supplementary Fig. 2 should be viewed as an illustrative example rather than a clinically useable tool. By pre-computing outcomes for clinically relevant patterns and enabling rapid retrieval of estimated cardiac event–free survival, the tool provides a conceptual framework for translating registry data into clinical decision support. As registry data are updated over time, prognostic estimates can be recalculated, allowing the model to reflect evolving outcomes and treatment practices. However, given the limited number of events and the resulting uncertainty in some estimates, particularly for sparsely represented profiles, the predictions should be interpreted with caution. Furthermore, this model has not undergone external validation, and its clinical applicability remains to be established.

### 4.4 Limitations

Several study limitations should be acknowledged. First, the relatively short follow-up period may have led to an underestimation of the true incidence of cardiac events, particularly those occurring over the longer term. Accordingly, the present findings primarily reflect short-term outcomes, and longer follow-up is required to fully assess long-term prognosis. In addition, the small number of observed events constrained statistical power and model precision. As a result, hazard estimates may be unstable, and the selection of predictors and cutoff values should be interpreted with caution and require further validation in larger cohorts with longer follow-up. For profiles with few or no observed patients, predicted estimates should be interpreted as model-based extrapolations rather than empirically supported observations. Second, although internal validation using bootstrapping and shrinkage techniques was performed to address potential overfitting, the model has not undergone external validation, and penalized regression methods were not applied. Therefore, its predictive performance and generalizability remain uncertain, and further validation using alternative modeling approaches is warranted. Third, as with all registry-based analyses, this study is subject to missing data, potential misclassification, and variability in the timing of clinical measurements relative to registry enrollment. In particular, the median interval from right heart catheterization to enrollment was 16.2 months. Therefore, baseline hemodynamic variables may not have fully reflected the patient's condition at time zero, and the model may have partly relied on non-contemporaneous predictors. Although the direction of associations was broadly consistent in the sensitivity analysis restricted to patients who underwent catheterization within 1 year before enrollment, further studies are needed to clarify whether these hemodynamic variables primarily function as real-time risk markers or as indicators of underlying disease severity. In addition, non-cardiovascular deaths were treated as censoring events rather than formal competing risks. Although this approach was adopted to maintain etiologic consistency of the composite endpoint, it represents a methodological limitation and may have affected estimation of cardiac event–free survival. Fourth, the proposed prognostic framework is based on patient characteristics upon registry enrollment and reflects outcomes under usual care. It does not account for dynamic changes over time or allow prediction of how prognosis may be modified by specific therapeutic interventions. Finally, the model was developed using data from a nationwide Japanese CHD-PH registry and is therefore primarily applicable to this population.

Taken together, this study illustrates a pragmatic approach to leveraging registry data for clinically accessible prognostic estimation in patients with CHD-PH. While the present findings are exploratory and should be interpreted with caution, the approach highlights the potential utility of integrating routinely collected clinical information into structured risk estimation. In particular, as registry data continue to accumulate over time, such frameworks may allow iterative refinement of prognostic estimates and better alignment with evolving patient characteristics and treatment practices. With the availability of high-quality regional or national registries, similar approaches may be adaptable to different clinical settings, providing context-specific insights that reflect local patient populations and healthcare environments.

## 5 Conclusion

This study introduces an exploratory, clinically oriented framework for estimating cardiac event–free survival in patients with CHD-PH using nationwide registry data. The findings demonstrate the feasibility of deriving exploratory, model-based prognostic estimates from routinely available clinical information; however, the framework should be considered as a proof-of-concept and requires further validation before clinical application.

## CRediT authorship contribution statement

**Taku Ishii:** Writing – original draft, Visualization, Software, Methodology, Formal analysis, Data curation, Conceptualization. **Tatsuhiko Anzai:** Writing – review & editing, Validation, Software, Methodology, Data curation, Conceptualization. **Keiko Uchida:** Conceptualization. **Susumu Hosokawa:** Writing – review & editing, Investigation, Conceptualization. **Naofumi F. Sumitomo:** Writing – review & editing, Investigation, Conceptualization. **Hidekazu Ishida:** Writing – review & editing, Investigation, Conceptualization. **Keiichi Hirono:** Writing – review & editing, Investigation. **Jun Muneuchi:** Writing – review & editing, Investigation. **Ayako Chida-Nagai:** Writing – review & editing, Investigation. **Ryo Inuzuka:** Writing – review & editing, Investigation. **Hirofumi Sawada:** Writing – review & editing, Investigation. **Sayo Suzuki:** Writing – review & editing, Investigation. **Jun Maeda:** Writing – review & editing, Investigation. **Hisaaki Aoki:** Writing – review & editing, Investigation. **Lisheng Lin:** Writing – review & editing, Investigation. **Takashi Murakami:** Writing – review & editing, Investigation. **Yusuke Nakano:** Writing – review & editing, Investigation. **Tatsuya Onishi:** Writing – review & editing, Investigation. **Takuya Wakamiya:** Writing – review & editing, Investigation. **Kei Inai:** Writing – review & editing, Investigation, Conceptualization. **Shinichi Takatsuki:** Writing – review & editing, Investigation, Conceptualization. **Atsushi Yao:** Writing – review & editing, Investigation. **Shigetoyo Kogaki:** Writing – review & editing, Supervision, Conceptualization. **Hiroyuki Fukushima:** Writing – review & editing, Supervision, Conceptualization. **Yuichi Tamura:** Writing – review & editing, Supervision, Resources, Conceptualization. **Kunihiko Takahashi:** Writing – review & editing, Validation, Software, Methodology, Data curation, Conceptualization. **Hiroyuki Yamagishi:** Writing – review & editing, Supervision, Conceptualization. **Shozaburo Doi:** Writing – review & editing, Supervision, Funding acquisition, Conceptualization.

## Declaration of generative AI and AI-assisted technologies in the manuscript preparation process

During the preparation of this manuscript, an artificial intelligence–based natural language processing tool (ChatGPT, OpenAI) was used to assist with drafting and revising English text and for checking typographical errors. The authors critically reviewed and edited all content, and the final version was reviewed by a professional native English editor. ChatGPT was also used to support discussions on statistical analysis coding strategies; however, all statistical analyses were independently designed, implemented, and interpreted by the authors.

## Funding statement

This work was supported by the 10.13039/100009619Japan Agency for Medical Research and Development (10.13039/100009619AMED) [grant number JP21ek0109546].

## Declaration of competing interest

The author is an Editorial Board Member/Editor-in-Chief/Associate Editor/Guest Editor for this journal and was not involved in the editorial review or the decision to publish this article.

The authors declare the following financial interests/personal relationships which may be considered as potential competing interests: H.Y. has received lecture fees for pulmonary hypertension–related lectures from AstraZeneca KK, Sanofi KK, and Janssen Pharma KK. All other authors declare no conflicts of interest.

## Data Availability

The original data underlying this study are derived from JACPHR. The data are not publicly available due to ethical restrictions. Access to the original registry data may be granted upon reasonable request and after review and approval by the JACPHR steering committee.
