# Supplementary material for: Registry-based estimation of cardiac event–free survival in congenital heart disease complicated by pulmonary hypertension: A nationwide registry study from Japan
Source: Int J Cardiol Congenit Heart Dis. 2026 Apr 19;25:100679. doi: 10.1016/j.ijcchd.2026.100679 (PMC13200114; doi:10.1016/j.ijcchd.2026.100679)
Supplement: Multimedia component 1 [file mmc1.pdf]

Supplementary Method 1. Inclusion and exclusion criteria for JACPHR

Inclusion Criteria:

1. No age restrictions.
2. Patients with any type of congenital heart disease (CHD) complicated by pulmonary hypertension (PH), who have neither undergone nor are scheduled to undergo surgery or catheter intervention that may improve PH within 6 months before or after the initial registration.
3. Diagnosis of PH is based on data from cardiac catheterization.
   3-1. The catheterization must have been performed on or after January 1, 2005 (it does not need to be the most recent study).
   3-2. PH is defined as
   mean pulmonary artery pressure (mPAP) > 20 mmHg or pulmonary vascular resistance index (PVRI) ≥ 3 Wood units·m².
4. Eligible patients include those with:
   4-1. Unrepaired shunt lesions (regardless of shunt size)
   4-2. Repaired shunt lesions (postoperative cases)
5. Two specific patient subgroups are also included under the following criteria:
   5-1. Special subgroup 1: Post-Glenn or Fontan procedure
   <Inclusion criteria for Special subgroup 1>
   Transpulmonary pressure gradient (TPG) > 6 mmHg or PVRI ≥ 3 Wood units·m²
   5-2. Special subgroup 2: Complex CHD with pulmonary atresia/stenosis and multiple major aortopulmonary collateral arteries (MAPCAs)
   <Inclusion criteria for Special subgroup 2>
   1. Pulmonary blood flow is supplied via a unified pathway (after unifocalization)
   2. Cardiac catheterization shows mPAP > 20 mmHg in at least one lung segment
6. Patients currently enrolled in other clinical trials are eligible.
7. For patients followed at multiple institutions, registration should be done by the facility where cardiac catheterization is regularly performed.
8. Patients are categorized as follows based on the interval between PH diagnosis (i.e., diagnostic catheterization) and consent date:
   Incident cases: Consent obtained within 3 months of diagnosis
   Prevalent cases: Consent obtained more than 3 months after diagnosis
   For prevalent cases, submit both the most recent catheterization data and—if available—data from the diagnostic catheterization prior to treatment initiation. If PH is first confirmed more than 6 months after surgery or catheter intervention, the date of that catheterization is considered the PH diagnosis date.

Exclusion Criteria:

1. Patients scheduled to undergo surgery or catheter intervention that may improve PH within approximately 6 months.
2. Patients who have undergone such surgery or intervention within the past 6 months.
3. Patients in whom PH is too unstable to allow for a definitive diagnosis.

Supplementary Method 2. Major Cardiac Diagnosis Used in JACPHR and Representative Conditions

The major cardiac diagnoses used in this study are classified as previously reported, with some modifications.

1. **Complex congenital heart disease**

   Hypoplastic Left Heart Syndrome

   Tricuspid Atresia

   Mitral Atresia

   Single Ventricle (Univentricular Atrioventricular Connection)

   Pulmonary Atresia with Intact Ventricular Septum

2. **Malposition of the great arteries**

   Complete Transposition of the Great Arteries (TGA)

   Congenitally corrected TGA

   Double-Outlet Right Ventricle (TGA type)

   Truncus Arteriosus

3. **Left-sided obstructive lesions**

   Coarctation/Interruption of the Aorta

   Congenital Aortic Stenosis (valvular, supra, infra)

   Congenital Aortic Regurgitation

4. **Right-sided obstructive lesions**

   Congenital Pulmonary Stenosis (valvular, supra, infra)

   Tetralogy of Fallot (TOF) with Pulmonary Stenosis, Pulmonary Atresia and Absent Pulmonary Valve

   Double-Outlet Right Ventricle (TOF type)

   Ebstein's anomaly

5. **Post-tricuspid shunts**

   Complete Atrioventricular Septal Defect

   Ventricular Septal Defect

   Patent Ductus Arteriosus

   Aortopulmonary Window

6. **Pre-tricuspid shunts, and others**

   Atrial Septal Defect / Patent Foramen Ovale

   Incomplete Atrioventricular Septal Defect

   Totally Anomalous Pulmonary Venous Connection or Drainage

   Partially Anomalous Pulmonary Venous Connection or Drainage

7. **Other**

   Cor triatriatum

   other

Supplementary Method 3. Clinical Classification of PH used in JACPHR.

The clinical classification of PH in this registry follows the statement proposed at the World Symposium held by NICE in 2018

Group 1: Pulmonary Arterial Hypertension (PAH) associated with CHD

Pre-capillary PH was defined by the simultaneous presence of mPAP > 20 mmHg, pulmonary artery wedge pressure (PAWP) ≤ 15 mmHg, and pulmonary vascular resistance (PVR) ≥ 3 Wood units. In the present study, because a large proportion of the cohort consisted of pediatric cases, we classified patients into Group 1 if they met the pediatric criteria—namely, mPAP > 20 mmHg, PAWP ≤ 15 mmHg, and PVRI ≥ 3 Wood units·m².
Cases classified as Group 1 are further categorized into one of the following four subtypes at the time of registration.

1. **Eisenmenger syndrome**
   Cases with a large systemic-to-pulmonary shunt, either intracardiac or extracardiac, in which pulmonary vascular resistance has become severely elevated—presumed to be irreversible—resulting in a reverse (pulmonary-to-systemic) or bidirectional shunt.
2. **Left-to-right shunts**
   Cases with a moderate-to-large systemic-to-pulmonary shunt accompanied by elevated PVR, but without the development of cyanosis. This includes both correctable and non-correctable lesions.
3. **PH with coincidental or small defects (coincidental PH).**
   Cases with markedly elevated pulmonary PVR despite having cardiac defects that are not typically associated with PH.
4. **Postoperative PH**
   Cases in which PH persists despite complete repair of congenital heart disease and the absence of a significant residual shunt. This includes patients whose PH initially improved but subsequently worsened again.

Group 2: PH due to left heart disease

Group 2 includes cases in which the cause of PH is attributed to left heart disease (post-capillary).
Examples include patients with pulmonary vein stenosis following TAPVR repair, as well as those with congenital mitral stenosis or aortic stenosis.

Group 3: PH due to lung diseases and/or hypoxia

Group 3 includes patients who have CHD but whose PH is primarily caused by underlying lung disease or chronic hypoxia.

Group 5: PH with unclear and/or multifactorial mechanisms

Group 5 includes patients complicated complex CHD with pulmonary atresia and major aortopulmonary collateral arteries (MAPCAs) who present elevated pulmonary pressure after MAPCA unifocalization and right ventricular outflow tract reconstruction (Segmental PH). It also includes single-ventricle patients with pulmonary vascular disease following Glenn or Fontan procedures (Fontan circulation).

Other

In this study, cases that could not be classified into Group 2, 3, or 5 and did not fulfill the diagnostic criteria for pulmonary arterial hypertension (PAH) were categorized as "Other."

Supplementary Method 4. Statistical analysis and prognostic modeling.

- **Model specification and missing data handling**

  Cox proportional hazards models were used to evaluate associations between candidate predictors and cardiac event–free survival. Analyses were conducted using complete-case data, excluding observations with missing values for variables included in the model; no imputation was performed due to the relatively small sample size and limited number of events. Model fit and proportional hazards assumptions were assessed using standard diagnostic procedures.

- **Internal validation and shrinkage**

  Given the limited number of events relative to the number of predictors, internal validation was performed using bootstrap resampling to assess potential overfitting and optimism in model performance. When optimism was identified, uniform shrinkage of regression coefficients was applied. Penalized regression methods were considered; however, given the exploratory nature of this study and the limited number of events, we prioritized model interpretability and applied bootstrap-based shrinkage as an alternative approach.

- **Subgroup and sensitivity analyses**

  Prespecified subgroup analyses were conducted to account for potential heterogeneity, including stratification by age category (children and adolescents vs adults), major CHD subtype (pre-tricuspid vs post-tricuspid shunt), and a sensitivity cohort restricted to patients who underwent right heart catheterization within 1 year prior to registry enrollment. In addition, a subgroup analysis restricted to patients without residual shunt was performed to evaluate a more homogeneous population.

- **Prediction framework and risk estimation**

  Based on the fitted Cox model, predicted cardiac event–free survival at 1, 2, and 3 years was estimated for predefined clinical profiles, along with corresponding 95% confidence intervals. For selected representative profiles, adjusted survival curves were generated, and predicted survival probabilities at 1 and 2 years were calculated. When applicable, shrinkage-adjusted survival estimates were also derived.

- **Clinical implementation and interpretation**

  To facilitate interpretation, the distribution of patients across clinical profiles was examined, and the number of patients corresponding to each profile was reported. Representative clinical scenarios were used to illustrate predicted outcomes, and a prototype clinical prediction tool was constructed to enable practical application of the model.

Supplementary Table 1. Distribution of cardiac events by PH classification and underlying cardiac diagnosis

	Total	With shunt	Without shunt	Left heart	Other
No. of patients / event	224 / 23	47 / 6	83 / 12	20 / 3	51 / 2
VSD	8	3	4	1	0
ASD	6	2	4	0	0
complete AVSD	2	0	2	0	0
TOF (PS or PA)	2	0	0	0	2
Aortic valve stenosis	1	0	1	0	0
AV valve anomalies	1	0	0	1	0
incomplete AVSD	1	0	1	0	0
TAPVR	1	0	0	1	0
other	1	1	0	0	0

Cardiac diagnoses among patients who experienced cardiac events during the study period, stratified by pulmonary hypertension classification. Tetralogy of Fallot with pulmonary atresia includes cases with segmental PH associated with major aortopulmonary collateral arteries. The total anomalous pulmonary venous return case represents postoperative pulmonary vein stenosis.

ASD, atrial septal defect; AVSD, atrioventricular septal defect; AV valve, atrioventricular valve; TAPVR, total anomalous pulmonary venous return; PA, pulmonary atresia; PH, pulmonary hypertension; PS, pulmonary stenosis; TOF, Tetralogy of Fallot

Supplementary Table 2. Sensitivity analysis using an alternative cutoff for pulmonary vascular resistance index (PVRI ≥5 WU·m²) in the Cox Proportional Hazards model

		Model 1<br>HR (95% CI)	Model 2<br>HR (95% CI)
Age at registration	≥18 years	1.09 (0.39–3.04)	0.87 (0.29–2.56)
Comorbidities	Trisomy 21	**3.57 (1.21–10.5)**	**3.48 (1.20–10.1)**
PH classification	With LR shunt	Ref	Ref
	Without LR shunt	1.83 (0.66–5.11)	1.97 (0.70–5.56)
	Left heart disease	1.73 (0.20–15.2)	2.08 (0.23–18.9)
	Other	1.28 (0.23–7.08)	1.09 (0.19–6.22)
PVRI at registration	PVRI ≥ 5 WU·m^2	**11.6 (2.91–45.9)**	**11.0 (2.70–44.5)**
CVP at registration	CVP > 10 mmHg	**3.25 (1.11–9.49)**	**3.50 (1.22–10.1)**
BNP at registration[a]	BNP > 50 pg/mL	1.80 (0.70–4.66)	1.87 (0.70–5.02)
Medication	No medication		Ref
	Monotherapy		1.22 (0.26–5.71)
	Combination therapy		2.19 (0.686–7.02

[a] BNP at registration: NT-proBNP values were used when BNP was not available (cutoff >400 pg/mL).

Cox proportional hazards analysis for cardiac event–free survival was repeated using an alternative cutoff for pulmonary vascular resistance index (PVRI ≥ 5 WU·m² instead of ≥ 6 WU·m² in the main analysis). The model was based on complete-case analysis and included the same covariates as in the primary model.

Hazard ratios are presented with 95% confidence intervals.

n indicates the number of patients, and events indicate the number of cardiac events.

CI, confidence interval; CVP, central venous pressure; HR, hazard ratio; PVRI, pulmonary vascular resistance index; WU, Wood units.

Bold values indicate $p < 0.05$.

Supplementary Table 3. Exploratory Subgroup Cox Proportional Hazards analyses for cardiac event–free survival

	**Child and adolescents**	**Adults**	**Pre-tricuspid shunt**	**Post-tricuspid shunt**	**CHD-PH without shunt**	**RHC within 1 year**
N / events	130 /10	60 / 11	51 / 7	65 / 9	87 / 12	81 / 10
Trisomy 21	**9.78 (2.40–39.8)**	NA (no events)	**27.1 (2.67–275)**	2.92 (0.64–13.4)	4.90 (1.43–16.7)	**7.66 (2.02–29.1)**
PVRI ≥6 WU·m^2	**17.2 (4.25–69.9)**	3.57 (0.45–28.6)	**41.0 (2.76–607)**	**23.0 (2.68–197)**	19.4 (4.06–93.1)	**9.79 (2.17–44.2)**
CVP > 10 mmHg	4.08 (0.74–22.5)	2.82 (0.44–9.70)	**13.0 (1.12–149)**	**8.44 (1.93–36.9)**	2.26 (0.56–9.09)	4.08 (0.76–22.0)

Exploratory subgroup Cox proportional hazards analyses were performed for cardiac event–free survival in the following subgroups: children and adolescents, adults, patients with pre-tricuspid and post-tricuspid shunt lesions, patients without shunt, and those who underwent right heart catheterization (RHC) within 1 year. Due to the limited number of events in each subgroup, the models were restricted to three clinically relevant covariates identified in the main analysis: trisomy 21, elevated pulmonary vascular resistance index (PVRI ≥6 WU·m^2), and elevated central venous pressure (CVP >10 mmHg). Children and adolescents were defined as individuals aged <18 years, and adults as those aged ≥18 years. Values are presented as hazard ratios (HRs) with 95% confidence intervals (CIs). “NA” indicates that the hazard ratio was not estimable due to the absence of events in the corresponding subgroup.

CHD, congenital heart disease; CI, confidence interval; CVP, central venous pressure; HR, hazard ratio; NA, not applicable; PH, pulmonary hypertension; PVRI, pulmonary vascular resistance index; RHC, right heart catheterization; WU, Wood units.

Supplementary Table 4. Distribution of patients across predictor combinations (profiles) used in the prognostic model

Number of patients per profile	Number of profiles	(%)
1	21	38.9
2	10	18.5
3	7	13.0
4	3	5.6
5	1	1.9
6	3	5.6
7	4	7.4
8	1	1.9
12	1	1.9
13	2	3.7
19	1	1.9

Each profile represents a unique combination of predictor variables included in the prognostic model. The table summarizes the distribution of the number of patients within each observed profile. A total of 54 distinct profiles were identified among 190 patients included in the complete-case analysis.

Supplementary Figure 1. Adjusted hazard ratios according to continuous PVRI and CVP based on the Cox proportional hazards model

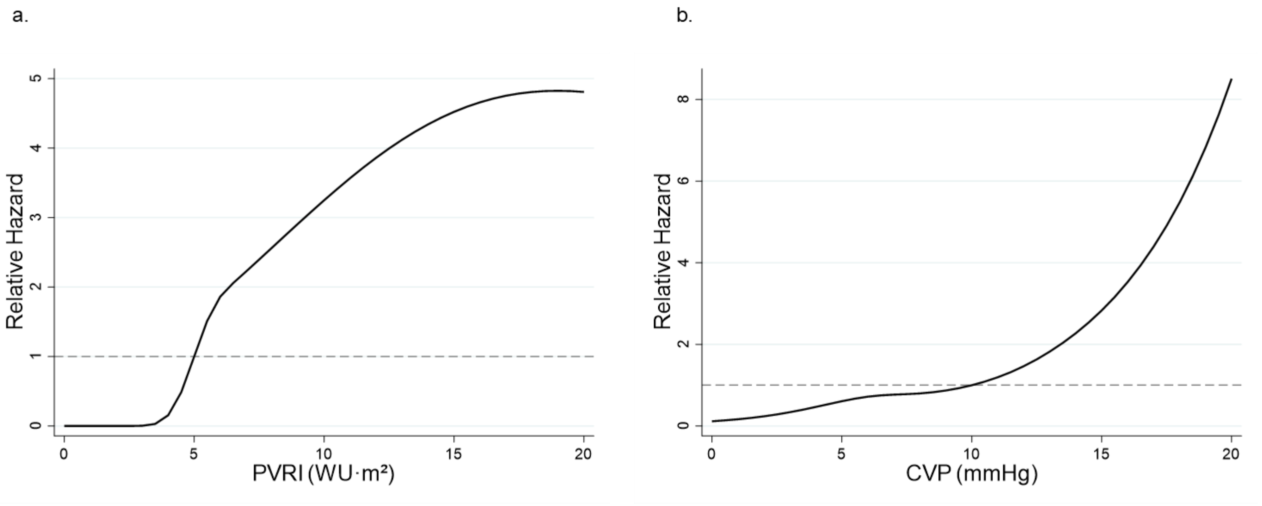

(a) Adjusted hazard ratios according to pulmonary vascular resistance index (PVRI).

(b) Adjusted hazard ratios according to central venous pressure (CVP).

Hazard ratios were estimated from the multivariable Cox proportional hazards model using restricted cubic splines. In each panel, the hazard ratio is expressed relative to a reference value (PVRI = 5 WU·m² in panel A and CVP = 10 mmHg in panel B).

Other covariates were fixed at representative values (pediatric patient, without trisomy 21 and normal BNP). The horizontal dashed line indicates a hazard ratio of 1.

Supplementary Figure 2. Prototype prediction tool for cardiac event–free survival in Japanese patients with CHD-PH

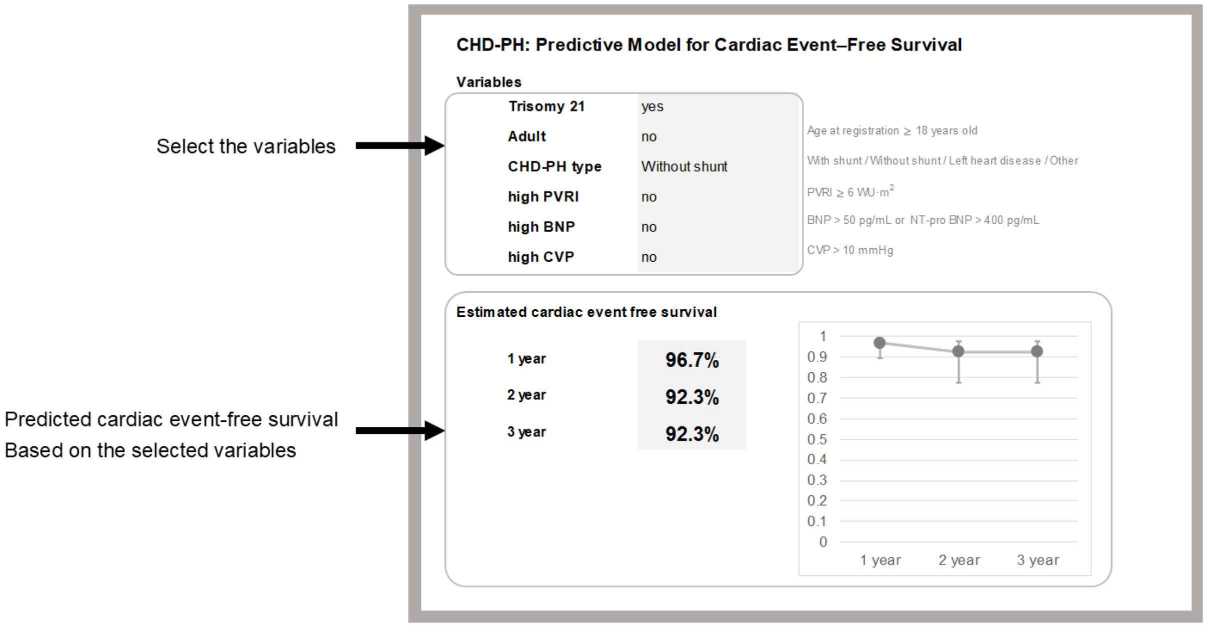

Using the Cox proportional hazards model, adjusted survival probabilities and corresponding 95% confidence intervals were pre-computed for all combinations of predictor variables and organized into a structured reference dataset. In this prototype, the user selects a specific clinical pattern, and the tool retrieves the corresponding predicted survival values, displaying the numerical estimates (predicted survival with 95% confidence intervals) and the adjusted survival curve. The figure shows the foundational reference sheet for this prediction tool. BNP, B-type natriuretic peptide; CHD, congenital heart disease; CVP, central venous pressure; NT-proBNP, N-terminal pro B-type natriuretic peptide; PH, pulmonary hypertension; PVRI, pulmonary vascular resistance index; WU, Wood units

Supplementary Acknowledgment. All participating institutions and principal investigators of the Japanese Association of CHD-PH Registry (JACPHR).

Institution	Investigators
Chiba Children's Hospital	Koji Higashi
Chiba Kaihin Municipal Hospital	Ryota Ebata, Shigeru Tateno
Chutoen General Medical Center	Satoshi Iwajima
Fukuoka Children's Hospital	Sayo Suzuki
Gunma Children's Medical Center	Kimiko Nakajima, Kentaro Ikeda
Hiroshima City Hiroshima Citizens Hospital	Naomi Nakagawa, Koichi Kataoka
Hokkaido University	Ayako Chida-Nagai, Atsuhito Takeda
Hyogo Prefectural Amagasaki General Medical Center	Takeaki Shirai, Kotaro Inakuma
Ibaraki Children's Hospital	Atsuko Shiono, Lisheng Lin
Institute of Science Tokyo	Taku Ishii, Susumu Hosokawa, Shozaburo Doi
Iwate Medical University	Satoshi Nakano, Hirofumi Saiki
Japan Community Healthcare Organization Kyushu Hospital	Daisuke Shimizu, Jun Muneuchi
Japanese Red Cross Saitama Hospital	Miki Karino
Jichi Medical University	Mitsuru Seki, Tomoyuki Kabutoya
Kanagawa Children's Medical Center	Takuya Wakamiya, Hideaki Ueda
Keio University	Naofumi F Sumitomo, Keiko Uchida, Hiroyuki Fukushima
Kitasato University	Yoichiro Hirata
Kurashiki Central Hospital	Tomohiro Hayashi, Kayo Ogino
Kyushu University	Yuichiro Hirata, Kenichiro Yamamura
Mie University	Hirofumi Sawada, Yoshihide Mitani
Miyazaki Prefectural Miyazaki Hospital	Toshinobu Ifuku, Shiro Adachi
Nagano Children's Hospital	Kota Takei, Kiyohiro Takigiku
Nagoya University	Soichiro Usui, Chiaki Goten
National Center for Child Health and Development	Shin Urata, Hiroshi Ono
National Hospital Organization Shikoku Medical Center for Children and Adults	Yuichi Miyagi, Tatsuya Omiya
Okayama university hospital	Maiko Kondo, Kenji Baba
Osaka General Medical Center	Shigetoyo Kogaki
Osaka Medical and Pharmaceutical University	Noriyasu Ozaki, Kanta Kishi
Osaka Women's and Children's Hospital	Toshiaki Aoki
Sakakibara Heart Institute	Tadahiro Yoshikawa, Hikoro Matsui
Shinshu University	Yukari Okuma, Mikiko Harada

Shizuoka Children’s Hospital	Norie Mitsushita
Showa University	Takanori Fujii, Toshiro Shinke
The University of Osaka Graduate School of Medicine	Hidekazu Ishida
The University of Tokyo	Ryo Inuzuka, Atsushi Yao
Toho University Omori Medical Center	Shinichi Takatsuki
Tokushima University	Yasunobu Hayabuchi, Kayoko Honma
Tokyo Metropolitan Children's Medical Center	Jun Maeda, Hiroyuki Yamagishi
Tokyo Women’s Medical University	Yoshiyuki Furutani, Kei Inai
Tottori University	Hitoshi Uemasu, Yoichi Mino
Tsuchiura Kyodo Medical Hospital	Tomohiro Watanabe
Ttuchiya General Hospital	Risa Morita, Kotaro Urayama
University of Toyama	Keiichi Hirono, Keijiro Ibuki
University of Tsukuba	Takashi Murakami, Yoshihiro Nozaki
University of Yamanashi	Yosuke Kono, Yohei Hasebe
Yokohama City University	Yusuke Nakano, Shun Kawai
